# Impact of cannabis abuse on the occurrence of stroke in young people: a systematic review and meta-analysis

**DOI:** 10.3389/fneur.2024.1426023

**Published:** 2024-10-22

**Authors:** Dongxue Liu, Liu Yang, Peiqi Liu, Yujiao Wang, Lan Gao

**Affiliations:** ^1^The First Hospital of Jilin University, Changchun, China; ^2^Department of Critical Care Medicine, Sir Run Run Shaw Hospital, Zhejiang University School of Medicine, Hangzhou, China; ^3^Department of Neurology, The First Hospital of Jilin University, Changchun, China

**Keywords:** youth stroke, cannabis abuse, occurrence, ischemic stroke, hemorrhagic stroke, risk factors

## Abstract

**Background:**

The occurrence of stroke in young people has risen significantly. This can easily lead to physical disabilities, swallowing difficulties, and cognitive impairment, among other issues, having a profound impact on families and society. Risk factors for stroke in young people differ from those for traditional stroke, with cannabis abuse emerging as a significant high-risk factor. However, the extent of the impact of cannabis abuse on the occurrence of stroke and the rate of disability in young people remains unclear. To clarify this issue and provide evidence supporting the primary prevention of stroke in young people, this systematic review and meta-analysis summarizes the latest findings from previous studies.

**Methods:**

A systematic search of PubMed, EMBASE, Cochrane Library, and Web of Science databases was conducted until April 2023. The review included observational studies comparing stroke risk estimates between cannabis abusers and non-users.

**Results:**

This review included six observational studies focusing on cannabis abuse, involving 119,284,152 participants. A significant association was found between cannabis abuse and an increased risk of stroke [OR = 1.14, 95% CI (1.08, 1.20)]. However, there was substantial heterogeneity among the studies (*I*^2^ = 89%, *p* < 0.001). After adjusting for confounders such as smoking and alcohol abuse, we found a stronger association between cannabis abuse and stroke in young adults [OR = 1.21, 95% CI (1.12, 1.29)]. Subgroup analyses revealed no significant difference in stroke risk between ischemic and hemorrhagic strokes (*p* = 0.43).

**Conclusion:**

The results of our systematic review and meta-analysis showed that cannabis abuse has a more significant effect on the occurrence of stroke in young people; however, it was not possible to distinguish whether cannabis abuse is more likely to cause ischemic or hemorrhagic stroke. Further research is needed to explore the impact of different drug types, dosages, and behaviors on stroke risk.

**Systematic review registration:**

https://www.crd.york.ac.uk/prospero/, Identifier CRD42023443261.

## Background

Youth stroke is defined as a stroke that occurs in individuals younger than 50 years of age (1), accounting for 10–15% of all stroke patients (2). Globally, the young population is considered the “backbone” of society. However, illness within this group not only impose a significant social burden but also reduce their personal quality of life. When young people return to their families following a stroke, they may also experience a sense of shame due to the subsequent lack of self-care abilities, body image disturbances, and other challenges, which will further hinder post-traumatic recovery (3). Some studies have reported that the etiology and risk factors in young patients are more diverse compared to older stroke patients, with hypertension, diabetes mellitus, coronary artery disease, excessive alcohol consumption, and smoking being definite risk factors (3). Low physical activity, hypertension, and smoking are considered high-risk factors, collectively accounting for 79% of stroke cases (4). Additionally, unknown risk factors (5), such as pregnancy, arterial entrapment, and illegal drug abuse, contribute to approximately 40% of cases (6). Strokes with no identifiable cause are classified as cryptogenic strokes, and younger patients have a higher incidence of this type, accounting for approximately 60% of cases (6). Therefore, identifying risk factors is key to preventing and reducing the incidence of stroke in young people.

Drug abuse refers to the non-medical use or overuse of drugs with dependence characteristics. The user’s dependence on such drugs and the compulsive pursuit of specific effects from these drugs leads to serious personal and public health issues as well as social problems (7, 8). The abuse of drugs such as cocaine, amphetamines, heroin, other opiates, and cannabis is becoming increasingly widespread, especially among young people in industrialized nations. It is estimated that heroin, cocaine, and other drugs can cause between 100,000 and 200,000 deaths annually. In addition to causing death, studies by Akasaki and Ohishi (7) have shown that drug abuse may be associated with an increased incidence of cardiovascular diseases such as acute coronary syndromes, arrhythmias, and aortic coarctation. Furthermore, Esse et al. (9) showed that drug abuse, especially overuse of opium, may also contribute to the development of stroke, with a high rate of resulting disability. Drug abuse may be a potential risk factor for the development of ischemic and hemorrhagic strokes in young people (10). However, some studies suggest that certain cases classified as illegal may, under specific circumstances, have protective effects on the body and could be used for the treatment of specific diseases after in-depth research and strict approval (11, 12). Therefore, the relationship between drug abuse and stroke risk remains unclear. In addition, it is not clear whether factors such as the type of drug abuse, duration of use, or frequency, are associated with the incidence of stroke and disability in younger oncology patients and other critically ill patients with a history of long-term use of analgesic and sedative drugs. Our study aimed to summarize the existing evidence regarding the impact of drug abuse on stroke occurrence in young people by conducting a systematic review with meta-analysis.

## Materials and methods

The present study has been prospectively registered in PROSPERO (CRD42021288033), and the reporting of our review follows the Preferred Reporting Items for Systematic Review and Meta-Analysis statement (PRISMA checklist and abstract checklist).

### Search strategies

We systematically searched PubMed, Embase, Web of Science, Cochrane Library, Cumulated Index in Nursing and Allied Health Literature (CINAHL), Wanfang Database, China National Knowledge Infrastructure (CNKI), and China Biology Medicine disc (CBMdisc). The search covered the period from the database’s inception to April 2023. The terms “young people,” “adults,” “youth,” “stroke,” “cerebrovascular stroke,” and “substance abuse” were used to construct the search strategy.

### Selection standards

The inclusion criteria are as follows: Patients aged 18–50 years with ischemic or hemorrhagic stroke; studies that reported the effect size of drug abuse on stroke occurrence and had a follow-up period of at least 1 year; cohort studies, case-control studies, or cross-sectional studies published in Chinese or English.

The exclusion criteria are as follows: Medically induced stroke or retinal infarction caused by any medical intervention; studies with inaccessible full text or duplicate publications; studies with incomplete data; studies rated as having low methodological quality.

### Data extraction and quality assessment

Literature screening was conducted independently by two researchers. Following the inclusion and exclusion criteria, data were extracted and cross-checked, and any disputes were resolved by negotiation and discussion or adjudicated by a third researcher. The retrieved literature was imported into NoteExpress, and the two researchers read titles and abstracts for initial screening. After initial screening, the literature was screened again by reading the full text to determine final inclusion. Information such as first author, publication date, country, study population, age, sample size, type of study, type of stroke, and type of substance abuse was extracted.

### Risk of bias assessment

The literature quality assessment was conducted independently by two trained researchers based on the literature risk of a bias assessment tool. The Newcastle Ottawa Scale (NOS) was used to assess the quality of both cohort and case-control studies. The cross-sectional research literature was evaluated using the evaluation criteria recommended by the Agency for Healthcare Research and Quality (AHRQ).

### Data analysis

The meta-analysis of risk factors was performed in this study using Stata17 software. A random-effects analysis was conducted to account for between-study heterogeneity. The results are presented as the odd ratio (OR) with a corresponding 95% confidence interval (CI). A *p*-value of <0.05 was considered statistically significant. Funnel plots were generated to assess publication bias in this study (13). Using Python software for weighted regression, the confounding factors of smoking and alcohol abuse were adjusted, and then the impact of cannabis abuse on young stroke occurrence was evaluated.

### Heterogeneity and subgroup analyses

We assessed statistical heterogeneity using chi-squared and *I*^2^ test statistics, considering chi^2^ <0.1 or *I*^2^ >50% as significant heterogeneity. Heterogeneity between studies was explored by conducting predefined subgroup analyses. The types of study included cohort, case-control, or cross-sectional studies, and two types of strokes were included: ischemic stroke and hemorrhagic stroke.

## Results

### Characteristics of the included studies

**Figure 1:**
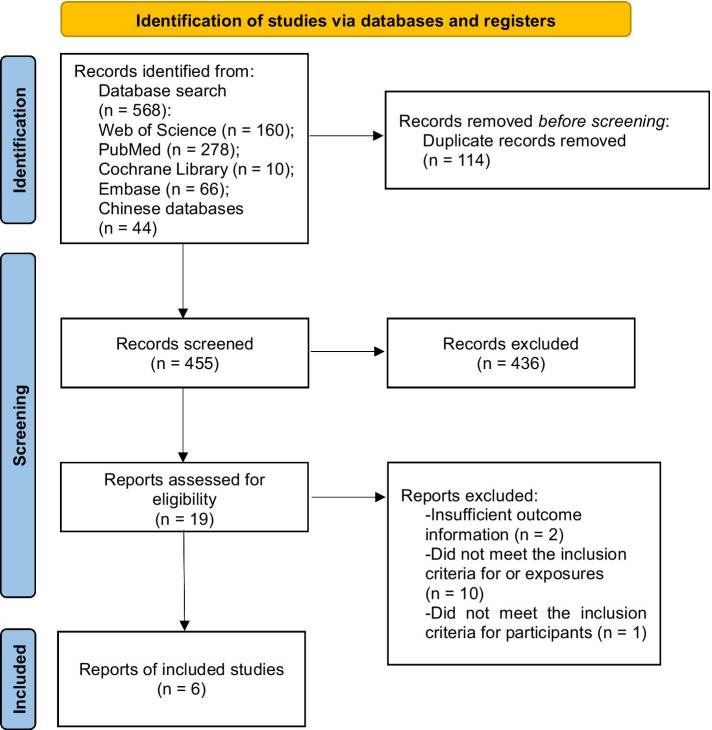
A flowchart of literature screening and the selection process.

A preliminary literature search was conducted for 569 articles. After eliminating duplicates, 455 articles remained, of which 436 articles were excluded because their titles and abstracts did not meet the requirements. The full texts of the remaining 19 articles were reviewed in detail, and 13 of them were excluded for not meeting the established criteria. Therefore, six articles were finally included (10, 14–18). Details of the quantitative analysis, and the process of literature screening are illustrated in Figure 1. Out of the six original papers included, four were cohort studies, one was case-control studies, and one was a cross-sectional study. The total number of stroke patients was 119,284,152, of whom 2,545,919 (2.13%) were younger patients, aged 18–50 years, with stroke onset associated with illicit drug abuse and a follow-up period of 1 year or more. The basic characteristics of the included literature are shown in Figure 1.

### Risk of bias

Cohort and case-control studies were evaluated using the NOS, with four cohort studies and one case-control study receiving a quality evaluation score of 6–8. Cross-sectional studies were evaluated based on the criteria recommended by AHRQ, with the literature scoring seven points. The evaluation results are shown in Table 1.

**Table 1:** Characteristics of the included literature.

Author, year published	Country	Study design	Sample size	Age (years)	Stroke type	Drug abuse type	Study time	Length of stay (years)	Literature quality
Rupak D, (2020) (15)	America	Cohort	34,857	18–49	IS	Cannabis	2007–2014	7	7
Kavelin R, (2016) (18)	America	Cohort	478,649	15–54	IS	Cannabis	2004–2011	7	6
Konark M, (2018) (17)	America	Cohort	103,357	15–54	IS/ICH	Cannabis	2004–2011	7	8
David AK, (1990) (10)	America	Case-control	214	15–44	IS/ICH	Cannabis	1979–1988		8
Dilini H, (2016) (16)	America	Cohort	7,455	20–64	TIA	Cannabis	1999–2002	1	8
Kavelin R, (2016) (14)	America	Cross-sectional	118,659,620	15–54	IS	Cannabis	2004–2011		7

### Publication bias

Publication bias was identified using a funnel plot and tested using Egger’s linear regression test (*p* = 0.227).

### Primary outcome

**Figure 2:**
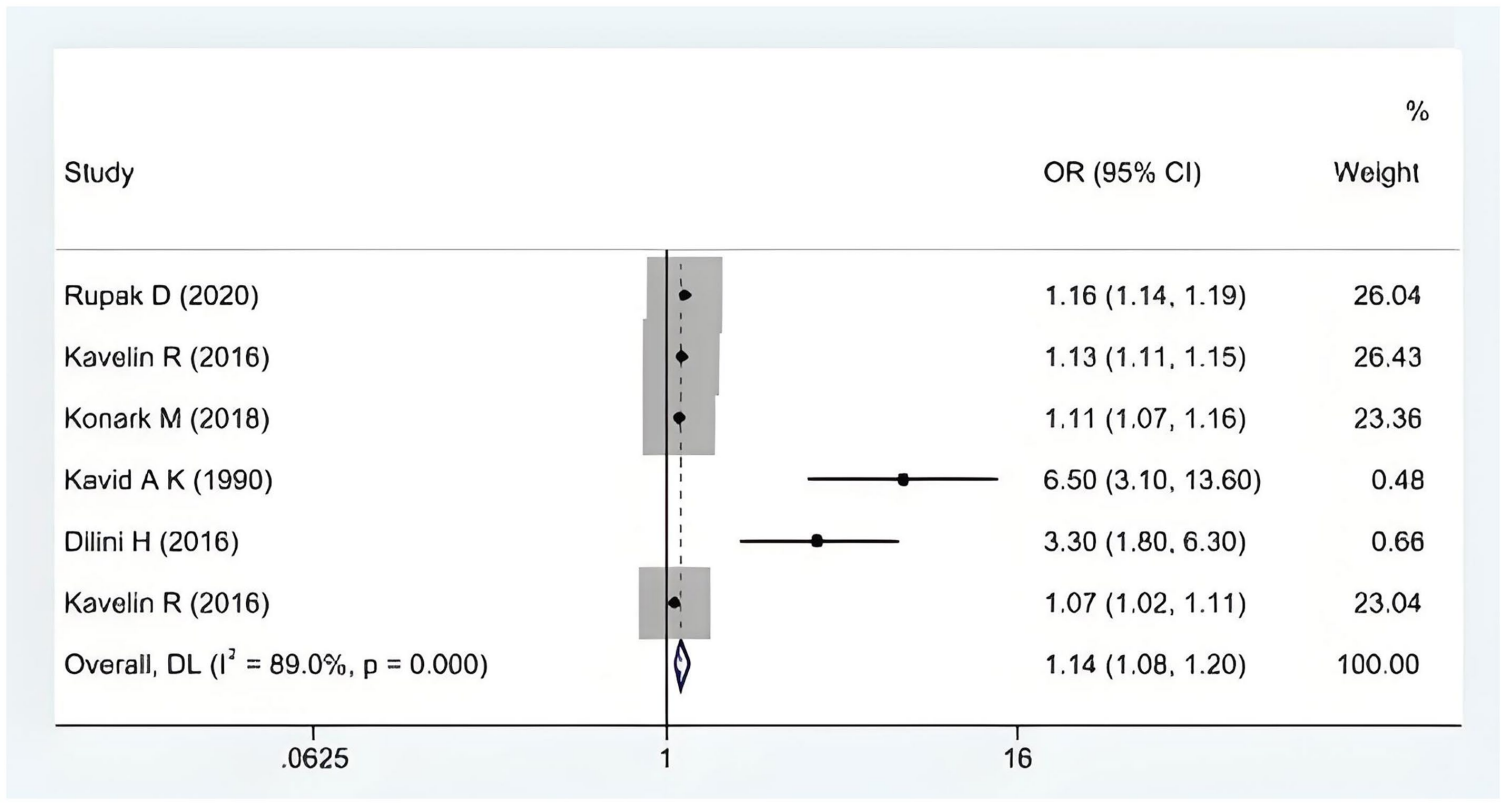
The impact of cannabis abuse on the occurrence of stroke in young people.

**Figure 3:**
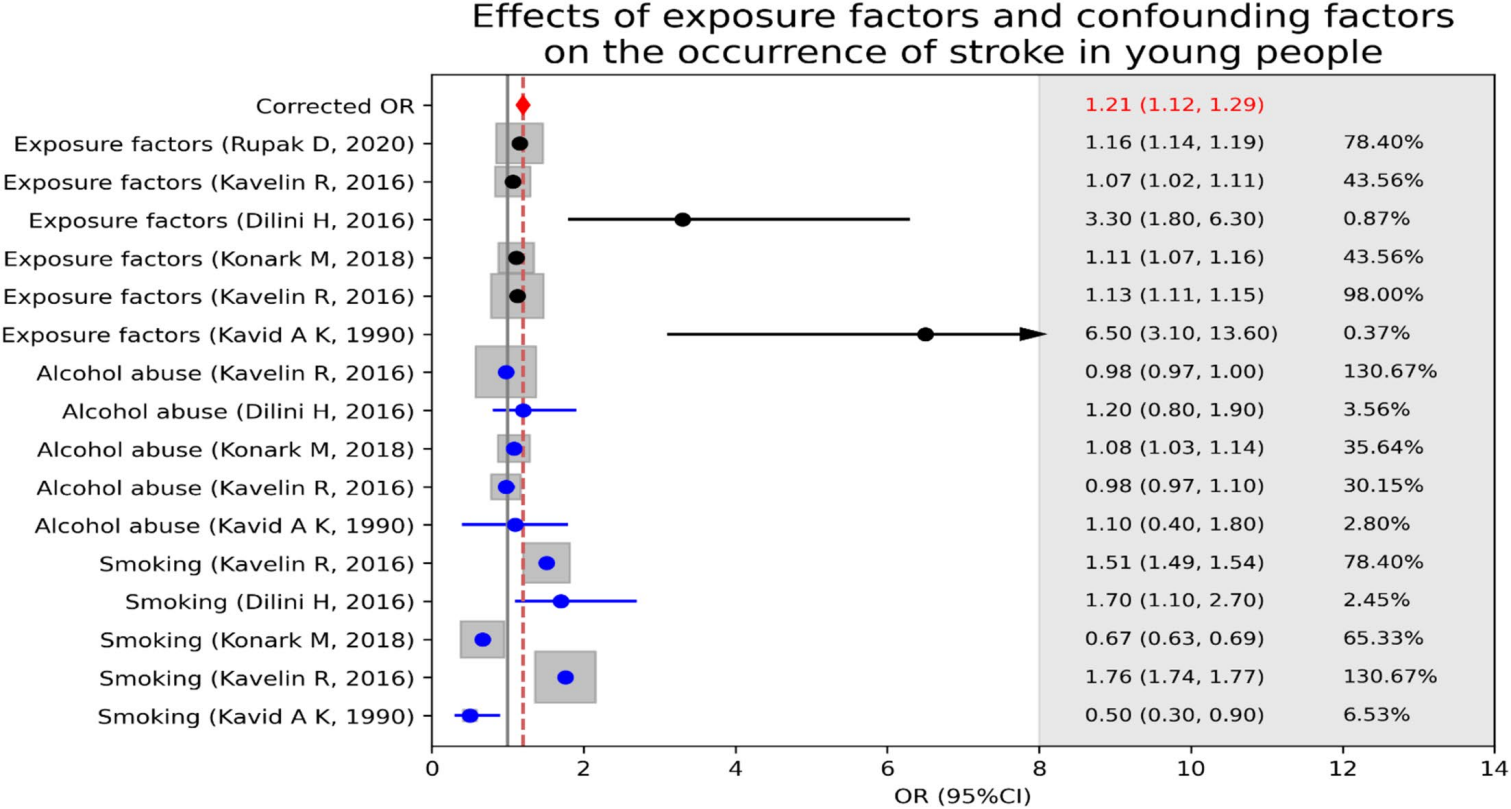
Effects of exposure factors (cannabis abuse) and confounding factors (smoking or alcohol abuse) on the occurrence of stroke in young people.

When confounding factors were not adjusted, cannabis abuse contributed to stroke incidence in young people [OR = 1.14, 95% CI (1.08, 1.20)], as shown in Figure 2. However, there was a large heterogeneity between the evidence (*I*^2^ = 89%, *p* < 0.001). After adjusting for confounders (smoking OR alcohol abuse), we found a stronger association between cannabis abuse and stroke in young adults [OR = 1.21, 95% CI (1.12, 1.29)]. These results indicate that cannabis abuse has a more significant effect on the occurrence of stroke in young people after excluding the interference of confounding factors, as shown in Figure 3.

### Sensitivity analysis and subgroup analysis

Sensitivity analysis was conducted by alternately excluding studies using Stata 17. The results of each study remained within the previous confidence intervals, and the original meta-analysis findings were not significantly altered by any individual study, indicating that the results were robust (Supplementary Figure S2).

**Figure 4:**
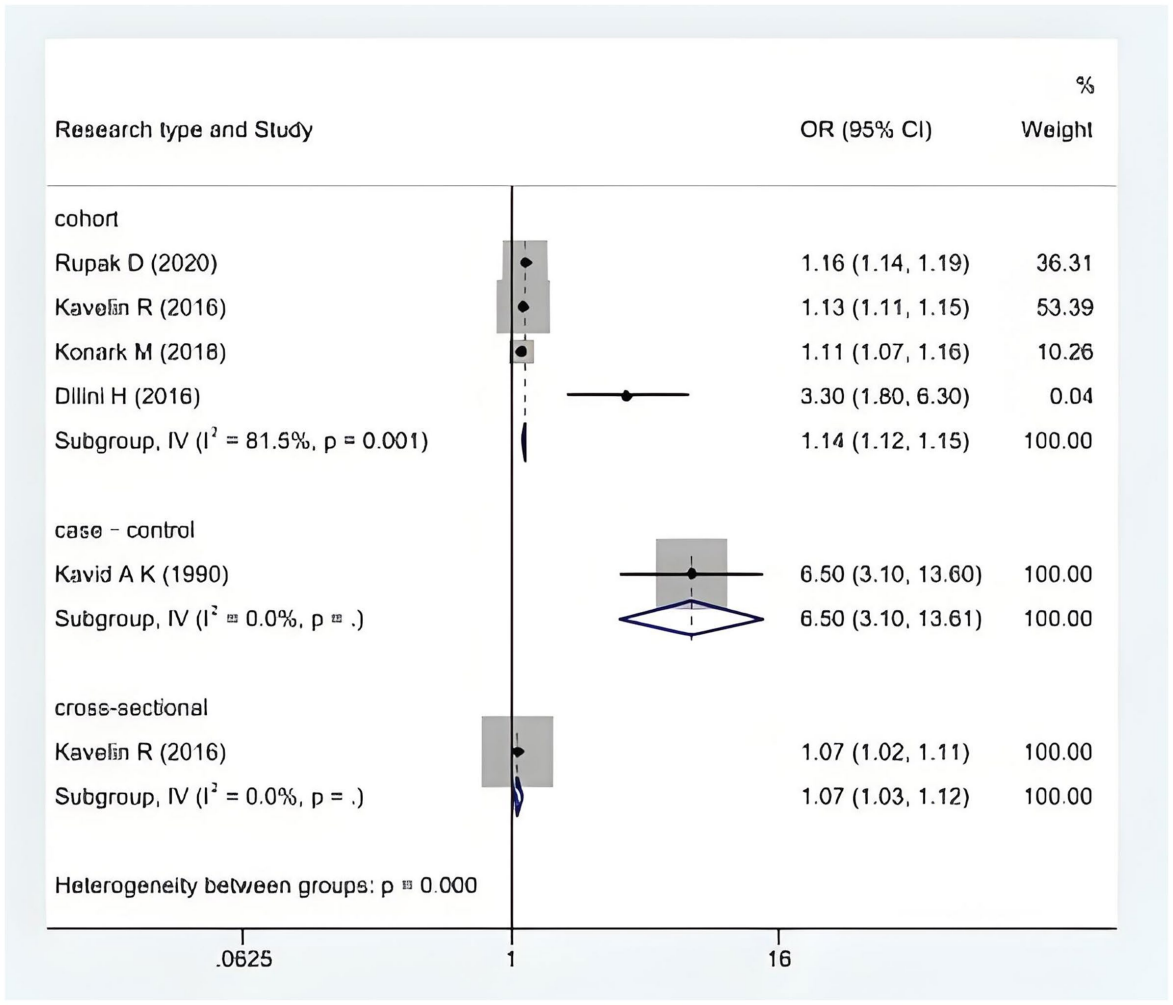
A forest plot with subgroup analysis according to the study type.

**Figure 5:**
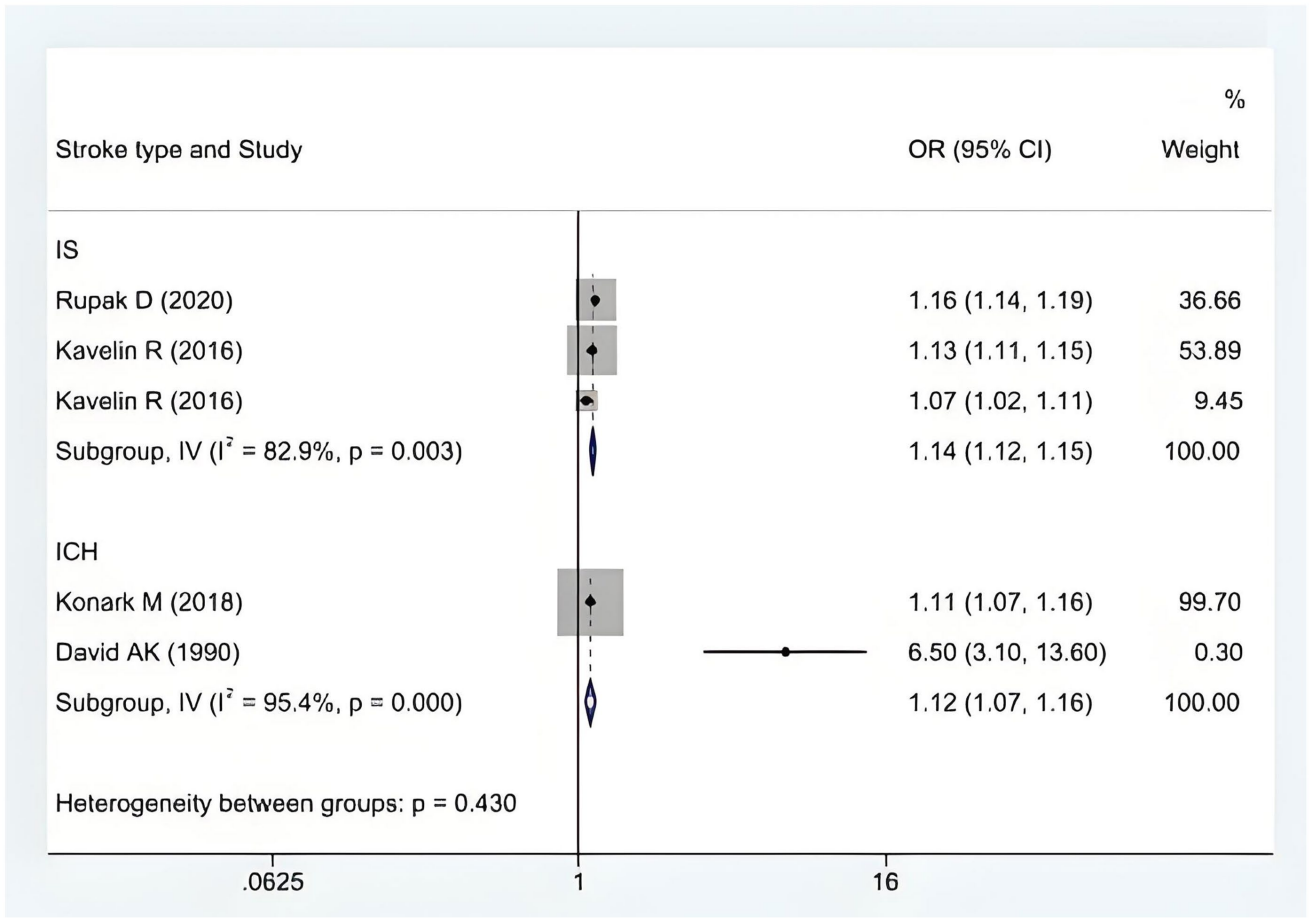
A forest plot with subgroup analysis according to the stroke type.

We performed a heterogeneity analysis, with subgroups analyzed by the type of study and the type of stroke, to assess whether significant subgroup differences existed. The analysis revealed a significant difference by study type (cohort vs. case-control vs. cross-sectional studies) (*p* < 0.001; see Figure 4). The six included studies showed that cannabis abuse had no significant difference between ischemic and hemorrhagic stroke. A meta-analysis using a random-effects model showed that the significant heterogeneity was due to the type of stroke, and the difference was statistically significant (*p* = 0.43; see Figure 5).

## Discussion

### Summary

The United Nations estimates that illicit drugs are used by approximately 200 million people (4.8% of the world’s population aged 15–64 years) each year (19). The occurence of stroke in young people is exponentially higher in areas with high rates of illicit drug abuse (20). However, the relationship between cannabis abuse and the incidence of stroke in young people has not been clearly identified in existing studies. We systematically pooled and analyzed evidence on the impact of cannabis abuse on the occurence of stroke in young patients, and a subgroup analysis, based on the stroke type, showed cannabis abuse has an impact on both ischemic and hemorrhagic stroke. This study provides important insights for the prevention and primary prevention of stroke in young people.

### Analysis of overall results

A total of six (10, 14–18) original articles (119,286,396 patients) were included in this study, and the findings were consistent with a study (10) investigating the etiology of stroke in young people, which showed that cannabis abuse could lead to an increased occurence of stroke in young people. This is due to the active ingredients in cannabis, such as tetrahydrocannabinol (THC), which can affect the contractile and diastolic functions of blood vessels. THC can cause blood vessels to spasm, reducing blood supply to the brain. Long-term or heavy use of cannabis can make blood vessels respond abnormally to normal physiological regulatory mechanisms, increasing the risk of blood vessel rupture or blockage (7), consistent with the findings of the article by Rose (21). A study by Cheng et al. (19) showed that in regions with a high frequency of illicit drug use, young people are an increased risk of stroke, with the incidence of stroke in young adults reported at 4.7%, such as in the Baltimore-Washington area (22). In contrast, our pooled data showed an incidence rate of only 2.13% in young people. This discrepancy may be attributed to factors such as regional differences, age, and sex. Therefore, these findings should be replicated in other cohorts, combining data from multiple population-based cohorts to improve the robustness of the results.

### Heterogeneity correlation analysis (analysis of subgroup outcomes)

This meta-analysis had a high degree of heterogeneity (*I*^2^ = 87.1%, *p* = 0.000), which may have stemmed from the type of study and the type of stroke.

### Cohort study key to highlighting causal relationship between cannabis abuse and stroke incidence in young adults

The articles included in this study were all observational studies, comprising four cohort studies, one case-control study, and one cross-sectional study. A common reason for high heterogeneity in observational studies is selection bias, which can make the exposed and non-exposed groups incomparable at baseline due to unaccounted confounding factors (23), such as illicit drug addiction. For example, heavy drug abusers—defined as individuals who have used illegal drugs more than 24 times in the past 2–6 months—have a higher incidence of non-fatal or transient stroke. In addition, the results of the cohort study showed more stability, demonstrating a stronger relationship between substance abuse and the onset of stroke in young stroke patients compared to the case-control and cross-sectional studies (*I*^2^ = 81.5%, *p* = 0.001).

### Cannabis abuse has an impact on both ischemic and hemorrhagic stroke

The increased heterogeneity in stroke types may be attributed to changes in vascular risk factors such as cerebral vasoconstriction and intracranial stenosis. A study by Desai et al. (16) suggested that cannabis intoxication may increase the risk of stroke through mechanisms such as orthostatic hypotension, as well as other cardiovascular abnormalities, such as diminished circulatory response to exercise, increased cerebrovascular resistance, and reduced cerebrovascular perfusion. Therefore, it could be beneficial to modify various lifestyle and health factors associated with stroke to reduce the rate of incidence.

### Strengths

Our meta-analysis and systematic review included cohort studies, case-control studies, and cross-sectional studies that summarized the relationship between young stroke patients and cannabis abuse from different perspectives and obtained similar results across studies. Despite high heterogeneity between studies, sensitivity and subgroup analyses showed relatively consistent and reliable results across the included studies.

### Limitations

Our meta-analysis had some limitations, with a small number of original articles included and a relatively high risk of bias. The variety of strokes and types of studies resulted in a high degree of heterogeneity between studies, which may reduce the perceived impact of cannabis abuse on stroke incidence in young people. All original literature included in this review were observational studies, meaning we cannot exclude the possibility that our findings may have been affected by residual confounders, and future studies should consider more variables or use a different design to minimize the influence of other factors such as demographic details, region, and sex. In addition, the relationship between the dose of illicit drugs and the severity and prognosis of stroke in young people needs to be further discussed depending on the clinical setting.

### Implications

Our study systematically reviewed a series of studies on the impact of cannabis abuse on the development of stroke in young people and found that patients with a history of cannabis abuse, especially young people, are at an increased risk of stroke. Therefore, it is crucial to conduct detailed inquiries about drug use history in this population and conduct urine and serum toxicological screening. Healthcare departments should enhance management efforts, prioritize the mental health of young people, disseminate knowledge on the dangers of illicit drugs such as opiates, and actively promote primary prevention in both urban and rural areas to mitigate the harmful effects of drug abuse.

## Conclusion

The results of our systematic review and meta-analysis showed that cannabis abuse has a significant effect on the occurrence of stroke in young people. However, it remains unclear whether cannabis abuse is more likely to cause ischemic or hemorrhagic stroke. Further research is needed to clarify the impact of different drug types, dosages, and usage patterns on stroke risk.

## Data Availability

The original contributions presented in the study are included in the article/Supplementary material, further inquiries can be directed to the corresponding authors.
