# Supplementary material for: Impact of cannabis abuse on the occurrence of stroke in young people: a systematic review and meta-analysis
Source: Front Neurol. 2024 Oct 22;15:1426023. doi: 10.3389/fneur.2024.1426023 (PMC11536518; doi:10.3389/fneur.2024.1426023)

**Supplementary material to the manuscript**

**The impact of drug abuse on the occurrence of stroke in young people: a systematic review and meta-analysis**

**Text S1:** Search strategy

**Figure S1:** Funnel plot

**Figure S2:** Sensitivity analysis

Text S1. Search strategy

Pubmed:

#1  **(Substance-Related Disorders[MeSH Terms]) OR (Substance-Related Disorders[Title/Abstract]) OR (Substance Related Disorder[Title/Abstract]) OR (Disorder, Substance Related[Title/Abstract]) OR (Disorders, Substance Related[Title/Abstract]) OR (Related Disorder, Substance[Title/Abstract]) OR (Related Disorders, Substance[Title/Abstract]) OR (Drug Use Disorders[Title/Abstract]) OR (Drug Use Disorder[Title/Abstract]) OR (Disorder, Drug Use[Title/Abstract]) OR (Organic Mental Disorders, Substance-Induced[Title/Abstract]) OR (Organic Mental Disorders, Substance Induced[Title/Abstract]) OR (Substance Abuse[Title/Abstract]) OR (Abuse, Substance[Title/Abstract]) OR (Substance Abuses[Title/Abstract]) OR (Substance Dependence[Title/Abstract]) OR (Dependence, Substance[Title/Abstract]) OR (Substance Addiction[Title/Abstract]) OR (Addiction, Substance[Title/Abstract]) OR (Chemical Dependence[Title/Abstract]) OR (Chemical Dependences[Title/Abstract]) OR (Dependence, Chemical[Title/Abstract]) OR (Dependences, Chemical[Title/Abstract]) OR (Drug Dependence[Title/Abstract]) OR (Dependence, Drug[Title/Abstract]) OR (Drug Addiction[Title/Abstract]) OR (Addiction, Drug[Title/Abstract]) OR (Prescription Drug Abuse[Title/Abstract]) OR (Abuse, Prescription Drug[Title/Abstract]) OR (Drug Abuse, Prescription[Title/Abstract]) OR (Substance Use[Title/Abstract]) OR (Substance Uses[Title/Abstract]) OR (Use, Substance[Title/Abstract]) OR (Drug Abuse[Title/Abstract]) OR (Abuse, Drug[Title/Abstract]) OR (Drug Habituation[Title/Abstract]) OR (Habituation, Drug[Title/Abstract]) OR (Substance Use Disorders[Title/Abstract]) OR (Disorder, Substance Use[Title/Abstract]) OR (Substance Use Disorder[Title/Abstract])**

#2 **(cocaine[MeSH Terms]) OR (cocaine[Title/Abstract]) OR (Cocaine Hydrochloride[Title/Abstract]) OR (Hydrochloride, Cocaine[Title/Abstract]) OR (Cocaine HCl[Title/Abstract]) OR (HCl, Cocaine[Title/Abstract])**

#3 **(**amphetamines **[MeSH Terms]) OR (**amphetamines **[Title/Abstract])**

#4 **(heroin[MeSH Terms]) OR (heroin[Title/Abstract]) OR (Diacetylmorphine[Title/Abstract]) OR (Diamorphine[Title/Abstract]) OR (Diamorf[Title/Abstract]) OR (Heroin Hydrochloride[Title/Abstract]) OR (Hydrochloride, Heroin[Title/Abstract]) OR (Diacetylmorphine Hydrochloride[Title/Abstract]) OR (Hydrochloride, Diacetylmorphine[Title/Abstract])**

#5  **(morphine[MeSH Terms]) OR (morphine[Title/Abstract]) OR (Morphia[Title/Abstract]) OR (Morphine Chloride[Title/Abstract]) OR (Chloride, Morphine[Title/Abstract]) OR (Morphine Sulfate[Title/Abstract]) OR (Sulfate, Morphine[Title/Abstract]) OR (SDZ 202-250[Title/Abstract]) OR (SDZ 202 250[Title/Abstract]) OR (SDZ 202250[Title/Abstract]) OR (SDZ202-250[Title/Abstract]) OR (SDZ202 250[Title/Abstract]) OR (SDZ202250[Title/Abstract]) OR (Morphine Sulfate (2:1), Pentahydrate[Title/Abstract]) OR (MS Contin[Title/Abstract]) OR (Contin, MS[Title/Abstract]) OR (Oramorph SR[Title/Abstract]) OR (Duramorph[Title/Abstract]) OR (Morphine Sulfate (2:1), Anhydrous[Title/Abstract])**

#6 (cannabis[MeSH Terms]) OR (cannabis[Title/Abstract]) OR (Cannabi[Title/Abstract]) OR (Hemp Plant[Title/Abstract]) OR (Hemp Plants[Title/Abstract]) OR (Plant, Hemp[Title/Abstract]) OR (Plants, Hemp[Title/Abstract]) OR (Marihuana[Title/Abstract]) OR (Marijuana[Title/Abstract]) OR (Cannabis indica[Title/Abstract]) OR (Cannabis sativa[Title/Abstract]) OR (Hemp[Title/Abstract]) OR (Hemps[Title/Abstract])

#7=#1 OR #2 OR #3 OR #4 OR #5 OR #6

#8  **(stroke[MeSH Terms]) OR (stroke[Title/Abstract]) OR (Cerebral stroke[Title/Abstract]) OR (Cerebrovascular stroke[Title/Abstract]) OR (Cerebrovascular accident[Title/Abstract]) OR (Ischemic stroke[Title/Abstract]) OR (Intracerebral hemorrhage[Title/Abstract])**

#9  **(Young Adult[MeSH Terms]) OR (Young Adult[Title/Abstract]) OR (Adult, Young[Title/Abstract]) OR (Adults, Young[Title/Abstract]) OR (Young Adults[Title/Abstract])**

#7 AND #8 AND #9

Web of science:

#1  **(AB=(Substance-Related Disorders) OR AB=(Substance Related Disorder) OR AB=(Disorder, Substance Related) OR AB=(Disorders, Substance Related) OR AB=(Related Disorder, Substance) OR AB=(Related Disorders, Substance) OR AB=(Drug Use Disorders) OR AB=(Drug Use Disorder) OR AB=(Disorder, Drug Use) OR AB=(Organic Mental Disorders, Substance-Induced) OR AB=(Organic Mental Disorders, Substance Induced) OR AB=(Substance Abuse) OR AB=(Abuse, Substance) OR AB=(Substance Abuses) OR AB=(Substance Dependence) OR AB=(Dependence, Substance) OR AB=(Substance Addiction) OR AB=(Addiction, Substance) OR AB=(Chemical Dependence) OR AB=(Chemical Dependences) OR AB=(Dependence, Chemical) OR AB=(Dependences, Chemical) OR AB=(Drug Dependence) OR AB=(Dependence, Drug) OR AB=(Drug Addiction) OR AB=(Addiction, Drug) OR AB=(Prescription Drug Abuse) OR AB=(Abuse, Prescription Drug) OR AB=(Drug Abuse, Prescription) OR AB=(Drug Abuse, Prescription) OR AB=(Substance Uses) OR AB=(Use, Substance) OR AB=(Drug Abuse) OR AB=(Abuse, Drug) OR AB=(Drug Habituation) OR AB=(Habituation, Drug) OR AB=(Substance Use Disorders) OR AB=(Disorder, Substance Use) OR AB=(Substance Use Disorder))**

**#2 (AB=(cocaine) OR AB=(Cocaine Hydrochloride) OR AB=(Hydrochloride, Cocaine) OR AB=(Cocaine HCl) OR AB=(HCl, Cocaine))**

#3 **AB=(amphetamines)**

#4 **(AB=(heroin) OR AB=(Diacetylmorphine) OR AB=(Diamorphine) OR AB=(Diamorf) OR AB=(Heroin Hydrochloride) OR AB=(Heroin Hydrochloride) OR AB=(Diacetylmorphine Hydrochloride) OR AB=(Hydrochloride, Diacetylmorphine))**

#5  **(AB=(morphine) OR AB=(Morphia) OR AB=(Morphine Chloride) OR AB=(Chloride, Morphine) OR AB=(Morphine Sulfate) OR AB=(Sulfate, Morphine) OR AB=(SDZ 202-250) OR AB=(SDZ 202 250) OR AB=(SDZ 202250) OR AB=(SDZ202-250) OR AB=(SDZ202 250) OR AB=(SDZ202250) OR AB=(Morphine Sulfate (2:1), Pentahydrate) OR AB=(MS Contin) OR AB=(Contin, MS) OR AB=(Oramorph SR) OR AB=(Duramorph) OR AB=(Morphine Sulfate (2:1), Anhydrous))**

#6  **(AB=(cannabis) OR AB=(Cannabi) OR AB=(Hemp Plant) OR AB=(Hemp Plants) OR AB=(Plant, Hemp) OR AB=(Plants, Hemp) OR AB=(Marihuana) OR AB=(Marijuana) OR AB=(Cannabis indica) OR AB=(Cannabis sativa) OR AB=(Hemp) OR AB=(Hemps))**

#7=#1 OR #2 OR #3 OR #4 OR #5 OR #6

#8 **(AB=(stroke) OR AB=(Cerebral stroke) OR AB=( Cerebrovascular stroke ) OR AB=(Cerebrovascular accident) OR AB=(Ischemic stroke) OR AB=(Intracerebral hemorrhage))**

**#9 (AB=(Young Adult) OR AB=(Adult, Young) OR AB=(Adults, Young) OR AB=(Young Adults))**

#7 AND #8 AND #9

**Cochrane Library:**

#1 **("Substance-Related Disorders"):ti,ab,kw OR ("Substance Related Disorder"):ti,ab,kw OR ("Disorder, Substance Related"):ti,ab,kw OR ("Disorders, Substance Related"):ti,ab,kw OR ("Related Disorder, Substance"):ti,ab,kw OR ("Related Disorders, Substance"):ti,ab,kw OR ("Drug Use Disorders"):ti,ab,kw OR ("Drug Use Disorder"):ti,ab,kw OR ("Disorder, Drug Use"):ti,ab,kw OR ("Organic Mental Disorders, Substance-Induced"):ti,ab,kw OR ("Organic Mental Disorders, Substance Induced"):ti,ab,kw OR ("Substance Abuse"):ti,ab,kw OR ("Abuse, Substance"):ti,ab,kw OR ("Substance Abuses"):ti,ab,kw OR ("Substance Dependence"):ti,ab,kw OR ("Dependence, Substance"):ti,ab,kw OR ("Substance Addiction"):ti,ab,kw OR ("Addiction, Substance"):ti,ab,kw OR ("Chemical Dependence"):ti,ab,kw OR ("Chemical Dependences"):ti,ab,kw OR ("Dependence, Chemical"):ti,ab,kw OR ("Dependences, Chemical"):ti,ab,kw OR ("Drug Dependence"):ti,ab,kw OR ("Dependence, Drug"):ti,ab,kw OR ("Drug Addiction"):ti,ab,kw OR ("Addiction, Drug"):ti,ab,kw OR ("Prescription Drug Abuse"):ti,ab,kw OR ("Abuse, Prescription Drug"):ti,ab,kw OR ("Drug Abuse, Prescription"):ti,ab,kw OR ("Substance Use"):ti,ab,kw OR ("Substance Uses"):ti,ab,kw OR ("Use, Substance"):ti,ab,kw OR ("Drug Abuse"):ti,ab,kw OR ("Abuse, Drug"):ti,ab,kw OR ("Drug Habituation"):ti,ab,kw OR ("Habituation, Drug"):ti,ab,kw OR ("Substance Use Disorders"):ti,ab,kw OR ("Disorder, Substance Use"):ti,ab,kw OR ("Substance Use Disorder")**

#2 **("cocaine"):ti,ab,kw OR ("Cocaine Hydrochloride"):ti,ab,kw OR ("Hydrochloride, Cocaine"):ti,ab,kw OR ("Cocaine HCl"):ti,ab,kw OR ("HCl, Cocaine")**

**#3 (“amphetamines”): ti,ab,kw**

**#4 ("heroin"):ti,ab,kw OR ("Diamorphine"):ti,ab,kw OR ("Diamorf"):ti,ab,kw OR ("Heroin Hydrochloride"):ti,ab,kw OR ("Hydrochloride, Heroin"):ti,ab,kw OR ("Diacetylmorphine Hydrochloride"):ti,ab,kw OR ("Hydrochloride, Diacetylmorphine")**

**#5 ("morphine"):ti,ab,kw OR ("Morphia"):ti,ab,kw OR ("Morphine Chloride"):ti,ab,kw OR ("Chloride, Morphine"):ti,ab,kw OR ("Morphine Sulfate"):ti,ab,kw OR ("Sulfate, Morphine"):ti,ab,kw OR ("SDZ 202-250"):ti,ab,kw OR ("Morphine Sulfate (2:1), Pentahydrate"):ti,ab,kw OR ("MS Contin"):ti,ab,kw OR ("Contin, MS"):ti,ab,kw OR ("Oramorph SR"):ti,ab,kw OR ("Duramorph"):ti,ab,kw OR ("Morphine Sulfate (2:1), Anhydrous")**

**#6 ("cannabis"):ti,ab,kw OR ("Cannabi"):ti,ab,kw OR ("Hemp Plant"):ti,ab,kw OR ("Hemp Plants"):ti,ab,kw OR ("Plant, Hemp"):ti,ab,kw OR ("Plants, Hemp"):ti,ab,kw OR ("Marihuana"):ti,ab,kw OR ("Marijuana"):ti,ab,kw OR ("Cannabis indica"):ti,ab,kw OR ("Cannabis sativa"):ti,ab,kw OR ("Hemp"):ti,ab,kw OR ("Hemps")**

**#7=#1 OR #2 OR #3 OR #4 OR #5 OR #6**

**#8 ("stroke"):ti,ab,kw OR ("Cerebral stroke** **"):ti,ab,kw OR ("Cerebrovascular stroke** **"):ti,ab,kw OR ("Cerebrovascular accident** **"):ti,ab,kw OR ("Ischemic stroke** **"):ti,ab,kw OR ("Intracerebral hemorrhage** **")**

**#9 ("Young Adult** **"):ti,ab,kw OR ("**Adult, Young**"):ti,ab,kw OR ("**Adults, Young**"):ti,ab,kw OR ("**Young Adults**")**

**#7 AND #8 AND #9**

Embase:

#1  **(Substance-Related Disorders[MeSH Terms]) OR (Substance-Related Disorders[Title/Abstract]) OR (Substance Related Disorder[Title/Abstract]) OR (Disorder, Substance Related[Title/Abstract]) OR (Disorders, Substance Related[Title/Abstract]) OR (Related Disorder, Substance[Title/Abstract]) OR (Related Disorders, Substance[Title/Abstract]) OR (Drug Use Disorders[Title/Abstract]) OR (Drug Use Disorder[Title/Abstract]) OR (Disorder, Drug Use[Title/Abstract]) OR (Organic Mental Disorders, Substance-Induced[Title/Abstract]) OR (Organic Mental Disorders, Substance Induced[Title/Abstract]) OR (Substance Abuse[Title/Abstract]) OR (Abuse, Substance[Title/Abstract]) OR (Substance Abuses[Title/Abstract]) OR (Substance Dependence[Title/Abstract]) OR (Dependence, Substance[Title/Abstract]) OR (Substance Addiction[Title/Abstract]) OR (Addiction, Substance[Title/Abstract]) OR (Chemical Dependence[Title/Abstract]) OR (Chemical Dependences[Title/Abstract]) OR (Dependence, Chemical[Title/Abstract]) OR (Dependences, Chemical[Title/Abstract]) OR (Drug Dependence[Title/Abstract]) OR (Dependence, Drug[Title/Abstract]) OR (Drug Addiction[Title/Abstract]) OR (Addiction, Drug[Title/Abstract]) OR (Prescription Drug Abuse[Title/Abstract]) OR (Abuse, Prescription Drug[Title/Abstract]) OR (Drug Abuse, Prescription[Title/Abstract]) OR (Substance Use[Title/Abstract]) OR (Substance Uses[Title/Abstract]) OR (Use, Substance[Title/Abstract]) OR (Drug Abuse[Title/Abstract]) OR (Abuse, Drug[Title/Abstract]) OR (Drug Habituation[Title/Abstract]) OR (Habituation, Drug[Title/Abstract]) OR (Substance Use Disorders[Title/Abstract]) OR (Disorder, Substance Use[Title/Abstract]) OR (Substance Use Disorder[Title/Abstract])**

#2 **(cocaine[MeSH Terms]) OR (cocaine[Title/Abstract]) OR (Cocaine Hydrochloride[Title/Abstract]) OR (Hydrochloride, Cocaine[Title/Abstract]) OR (Cocaine HCl[Title/Abstract]) OR (HCl, Cocaine[Title/Abstract])**

#3 **(**amphetamines **[MeSH Terms]) OR (**amphetamines **[Title/Abstract])**

#4 **(heroin[MeSH Terms]) OR (heroin[Title/Abstract]) OR (Diacetylmorphine[Title/Abstract]) OR (Diamorphine[Title/Abstract]) OR (Diamorf[Title/Abstract]) OR (Heroin Hydrochloride[Title/Abstract]) OR (Hydrochloride, Heroin[Title/Abstract]) OR (Diacetylmorphine Hydrochloride[Title/Abstract]) OR (Hydrochloride, Diacetylmorphine[Title/Abstract])**

#5  **(morphine[MeSH Terms]) OR (morphine[Title/Abstract]) OR (Morphia[Title/Abstract]) OR (Morphine Chloride[Title/Abstract]) OR (Chloride, Morphine[Title/Abstract]) OR (Morphine Sulfate[Title/Abstract]) OR (Sulfate, Morphine[Title/Abstract]) OR (SDZ 202-250[Title/Abstract]) OR (SDZ 202 250[Title/Abstract]) OR (SDZ 202250[Title/Abstract]) OR (SDZ202-250[Title/Abstract]) OR (SDZ202 250[Title/Abstract]) OR (SDZ202250[Title/Abstract]) OR (Morphine Sulfate (2:1), Pentahydrate[Title/Abstract]) OR (MS Contin[Title/Abstract]) OR (Contin, MS[Title/Abstract]) OR (Oramorph SR[Title/Abstract]) OR (Duramorph[Title/Abstract]) OR (Morphine Sulfate (2:1), Anhydrous[Title/Abstract])**

#6 (cannabis[MeSH Terms]) OR (cannabis[Title/Abstract]) OR (Cannabi[Title/Abstract]) OR (Hemp Plant[Title/Abstract]) OR (Hemp Plants[Title/Abstract]) OR (Plant, Hemp[Title/Abstract]) OR (Plants, Hemp[Title/Abstract]) OR (Marihuana[Title/Abstract]) OR (Marijuana[Title/Abstract]) OR (Cannabis indica[Title/Abstract]) OR (Cannabis sativa[Title/Abstract]) OR (Hemp[Title/Abstract]) OR (Hemps[Title/Abstract])

#7=#1 OR #2 OR #3 OR #4 OR #5 OR #6

#8  **(stroke[MeSH Terms]) OR (stroke[Title/Abstract]) OR (Cerebral stroke[Title/Abstract]) OR (Cerebrovascular stroke[Title/Abstract]) OR (Cerebrovascular accident[Title/Abstract]) OR (Ischemic stroke[Title/Abstract]) OR (Intracerebral hemorrhage[Title/Abstract])**

#9  **(Young Adult[MeSH Terms]) OR (Young Adult[Title/Abstract]) OR (Adult, Young[Title/Abstract]) OR (Adults, Young[Title/Abstract]) OR (Young Adults[Title/Abstract])**

#7 AND #8 AND #9

CNKI

(SU%='药物滥用' OR SU%='物质相关性障碍' OR SU%='药物依赖' OR SU%='药瘾' OR SU%='药物成瘾' OR SU%='物质应用所致精神障碍' OR SU%='药物使用所致精神障碍' OR SU%='物质性器质性精神障碍' OR SU%='物质滥用' OR SU%='物质依赖' OR SU%='物质成瘾' OR SU%='物质相关性障碍') AND (SU% ='青年' OR SU%='青少年') AND (SU% ='脑卒中' OR SU%='卒中' OR SU%='脑血管意外' OR SU%='脑中风' OR SU%=' CVA(脑血管意外)' OR SU%=' CVAs(脑血管意外)' OR SU%='脑血管中风' OR SU%='急性中风' OR SU%='急性卒中' OR SU%='急性脑血管意外' OR SU%='急性脑卒中')

Wanfang Database

(主题='药物滥用' OR 主题='物质相关性障碍' OR 主题='药物依赖' OR 主题='药瘾' OR 主题='药物成瘾' OR 主题='物质应用所致精神障碍' OR 主题='药物使用所致精神障碍' OR 主题='物质性器质性精神障碍' OR 主题='物质滥用' OR 主题='物质依赖' OR 主题='物质成瘾' OR 主题='物质相关性障碍') AND (主题='青年' OR 主题='青少年') AND (主题='脑卒中' OR 主题='卒中' OR 主题='脑血管意外' OR 主题='脑中风' OR 主题=' CVA(脑血管意外)' OR 主题=' CVAs(脑血管意外)' OR 主题='脑血管中风' OR 主题='急性中风' OR 主题='急性卒中' OR 主题='急性脑血管意外' OR 主题='急性脑卒中')

CBM

#1 "药物滥用"[全部字段:智能] OR "物质相关性障碍"[全部字段:智能] OR "药物依赖"[全部字段:智能] OR "药瘾"[全部字段:智能] OR "药物成瘾"[全部字段:智能] OR "物质应用所致精神障碍"[全部字段:智能] OR "药物使用所致精神障碍"[全部字段:智能] OR "物质性器质性精神障碍"[全部字段:智能] OR "物质滥用"[全部字段:智能] OR "物质依赖"[全部字段:智能] OR "物质成瘾"[全部字段:智能]

#2 "青年"[全部字段:智能] OR "青少年"[全部字段:智能]

#3 "脑卒中"[全部字段:智能] OR "卒中"[全部字段:智能] OR "脑血管意外"[全部字段:智能] OR "脑中风"[全部字段:智能] OR " CVA(脑血管意外)"[全部字段:智能] OR " CVAs(脑血管意外)"[全部字段:智能] OR "脑血管中风"[全部字段:智能] OR "急性中风"[全部字段:智能] OR "急性卒中"[全部字段:智能] OR "急性脑血管意外"[全部字段:智能] OR "急性脑卒中"[全部字段:智能]

#4= #1 AND #2 AND #3

Figure S1: Funnel plot


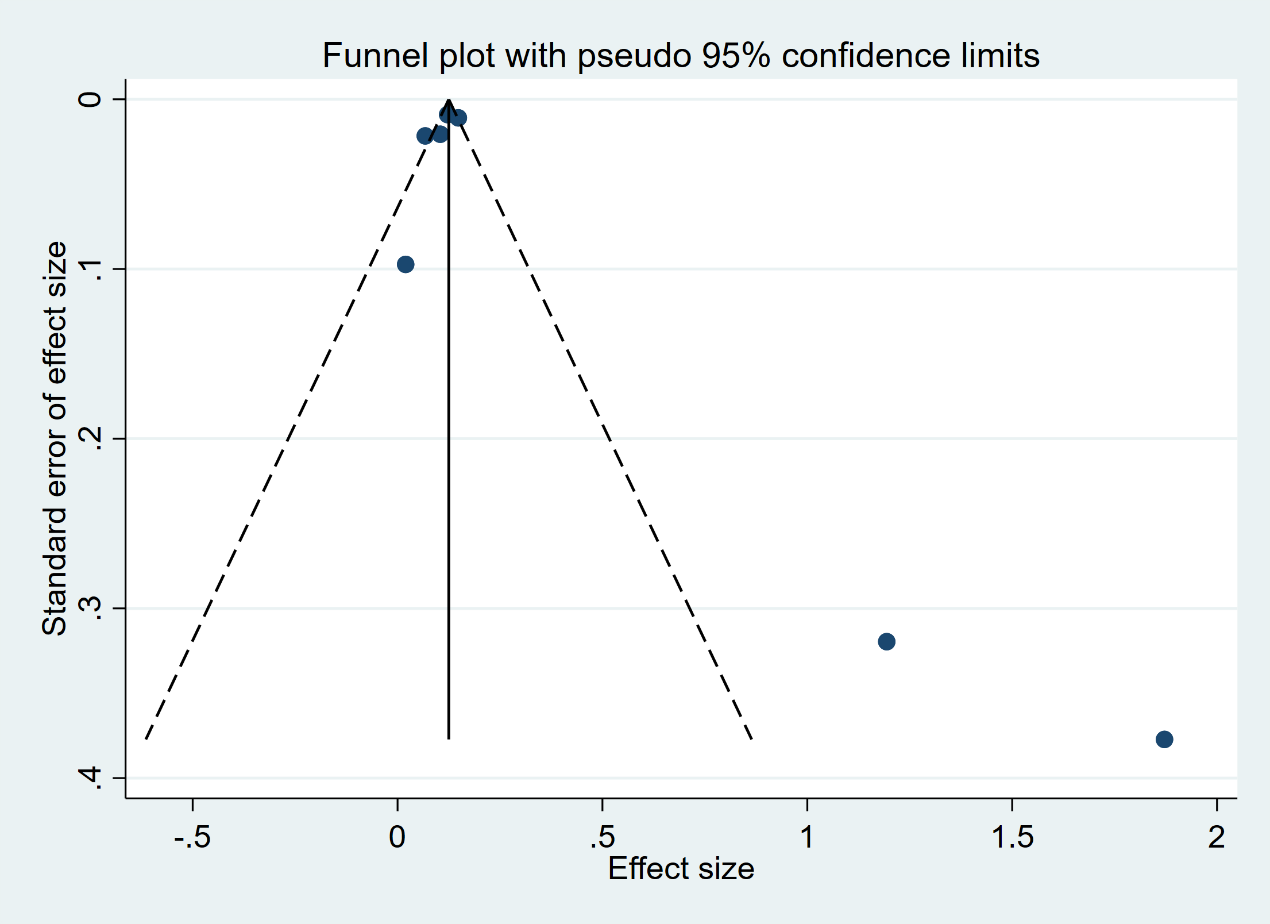


Figure S2**:** Sensitivity analysis


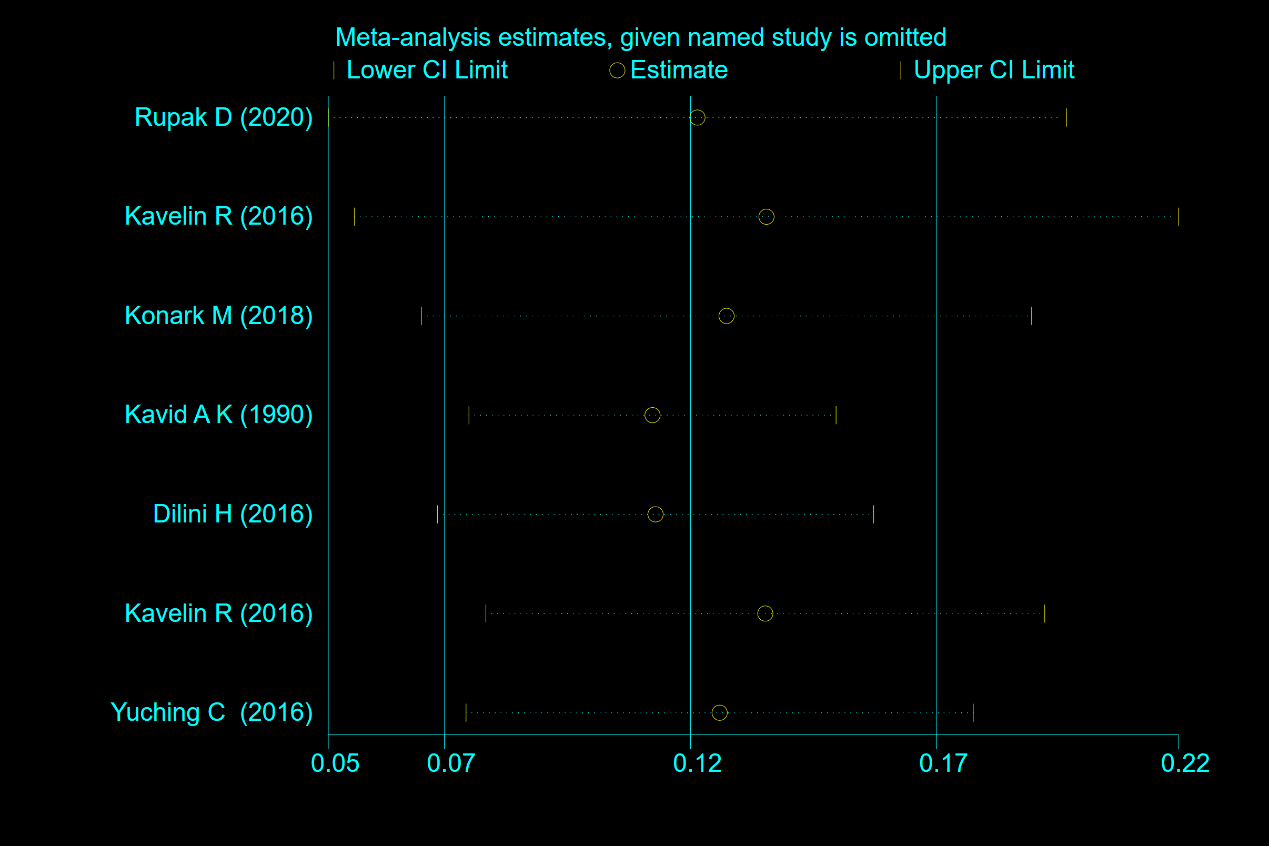

Supplement: Supplementary file 1 [file Data_Sheet_1.docx]
